# Fucoidan and Alginate from the Brown Algae *Colpomenia sinuosa* and Their Combination with Vitamin C Trigger Apoptosis in Colon Cancer

**DOI:** 10.3390/molecules27020358

**Published:** 2022-01-06

**Authors:** Reem Al Monla, Zeina Dassouki, Nouha Sari-Chmayssem, Hiba Mawlawi, Hala Gali-Muhtasib

**Affiliations:** 1 AZM Center for Research in Biotechnology and Its Applications, Laboratory of Applied Biotechnology (LBA3B), Doctoral School for Sciences and Technology, Lebanese University, Tripoli 1300, Lebanon; reem.el-monla@umontpellier.fr (R.A.M.); zeina.dassouki@ul.edu.lb (Z.D.); nouha.sari@hotmail.com (N.S.-C.); hiba.mawlawi@ul.edu.lb (H.M.); 2 Department of Biology, American University of Beirut, Riad El Solh, Beirut 1107 2020, Lebanon; 3 Center for Drug Discovery, American University of Beirut, Riad El Solh, Beirut 1107 2020, Lebanon

**Keywords:** fucoidan, alginate, apoptosis, reactive oxygen species, human colon cancer cells

## Abstract

Brown seaweeds are producers of bioactive molecules which are known to inhibit oncogenic growth. Here, we investigated the antioxidant, cytotoxic, and apoptotic effects of two polysaccharides from the brown algae *Colpomenia sinuosa*, namely fucoidan and alginate, in a panel of cancer cell lines and evaluated their effects when combined with vitamin C. Fucoidan and alginate were isolated from brown algae and characterized by HPLC, FTIR, and NMR spectroscopy. The results indicated that highly sulfated fucoidans had higher antioxidant and cytotoxic effects than alginate. Human colon cancer cells were the most sensitive to the algal treatments, with fucoidan having an IC_50_ value (618.9 µg/mL^−^^1^) lower than that of alginate (690 µg/mL^−^^1^). The production of reactive oxygen species was increased upon treatment of HCT-116 cells with fucoidan and alginate, which suggest that these compounds may trigger cell death via oxidative damage. The combination of fucoidan with vitamin C showed enhanced effects compared to treatment with fucoidan alone, as evidenced by the significant inhibitory effects on HCT-116 colon cancer cell viability. The combination of the algal polysaccharides with vitamin C caused enhanced degeneration in the nuclei of cells, as evidenced by DAPI staining and increased the subG1 population, suggesting the induction of cell death. Together, these results suggest that fucoidan and alginate from the brown algae *C. sinuosa* are promising anticancer compounds, particularly when used in combination with vitamin C.

## 1. Introduction

The marine environment is an exceptionally diverse reservoir comprised of nearly 250,000 species that produce numerous secondary compounds with extraordinary chemical and pharmacological effects [1]. The growing interest in marine macroalgae as a sustainable source of natural molecules is mainly due to their therapeutic effects against cancer [2]. Unlike toxic conventional chemotherapies, these marine products exert minimal side effects on healthy tissues [2]. In recent years, there has been growing interest in the polysaccharides present in cell walls of brown algae, including laminarins, alginates, and fucoidans, as these compounds have high potential for biological applications in functional foods as well as cosmeceutical and pharmaceutical bioproducts [3].

Fucoidans found in brown seaweeds cover a family of sulfated fucose-rich polysaccharides. They are made up of a backbone of (1→3) and/or (1→4) α-linked L-fucopyranose units [4]. Owing to their biofunctional properties, fucoidans are potent antitumor agents with numerous pharmaceutical applications [5]. Fucoidans have shown strong cytotoxicity against different cancer cell lines, including breast cancer (MCF-7), human skin melanoma (SK-MEL-28), and colon cancer (HCT-15, DLD1, WiDr) cells [6]. Fucoidans are also known to significantly reduce the toxicity of chemotherapy in patients with advanced and recurrent cancers [7] and are used as adjuvants in complex cancer treatments. Interestingly, dietary fucoidans and those from other brown algal species were found to enhance the anticancer effects of the chemotherapeutic drugs tamoxifen and lapatinib when used against a wide range of cancer cells and in mouse models of cancer [8,9].

Alginates are one of the most exploited natural polysaccharides for manufacturing value-added products and are widely used in the pharmaceutical industry [10,11], Alginates are linear copolymers made of β-D-mannuronic acid (M) and α-L-guluronic acid (G) bound in blockwise arrangements [12]. They have been shown to inhibit many cell lines including cervical (HeLa), hepatocellular (HepG2), breast (MDA-MB-231), and colon (HCT-116 and CaCo2) cancer cells [13,14,15]. Alginates were also found to protect human intestinal cells from carcinogenesis via their antioxidant effects and their ability to chelate heavy metals and toxins [16], and to act in combination with chitosan, bortezomib, and 5-Fluorouracil to inhibit cell proliferation [17,18,19].

Combination therapy of natural molecules is considered to be a promising strategy to boost the therapeutic efficacy of chemotherapeutics as well as reduce their toxicity and side effects [20]. High-dose vitamin C is safely employed as a complementary treatment against colon cancer [21]. Additionally, the combination of vitamin C with multiple standard drugs and natural molecules resulted in significant inhibitory effects against cancer cell growth and proliferation [22,23].

Studies on the Lebanese algae against cancer are very limited, and they mostly rely on colorimetric methods [24,25,26,27]. *C. sinuosa* is a brown algae that was previously investigated by our team, and the results confirmed an elevated carbohydrate content in the tissues of this species [28]. The cytotoxic potential of fucoidans and alginates of *C. sinuosa* on different cancer cells have never been examined, and the effects of their combination with vitamin C remains unknown. Thus, we studied the chemical properties as well as the antitumor and apoptotic potency of fucoidan and alginate from *C. sinuosa* alone and in combination with vitamin C against different cancer cell lines including colon (HCT-116), breast (MCF 7), and ovarian (HeLa) cancer cell lines.

## 2. Results

### 2.1. Physicochemical Properties and Monosaccharide Composition of Fucoidan and Alginate

The physicochemical characteristics of the extracted fucoidan and alginate of *C. sinuosa* are presented in Table 1. There was very low percentage of protein and phenol contaminants in the preparation of the isolated polysaccharides. To determine the degree of sulfation, the barium-chloride method was adopted, and the results showed that fucoidan was highly sulfated (18.8%) more than alginate (5.53%). Moreover, the percentage of D-glucuronic acid was much higher in alginate (50%) than fucoidan (3.8%) (Table 1).

The monosaccharide composition of the extracted polysaccharides showed that fucose is the main sugar present in fucoidan (67.4%) (Table 2). We observed large differences in the relative proportions of the different monosaccharides in fucoidan and alginate polymers. The HPLC spectra of both fucoidan and alginate are shown in Supplementary Figures S1 and S2.

### 2.2. Brown Algal Polysaccharides Structural Analysis by FTIR

**Figure 1:**
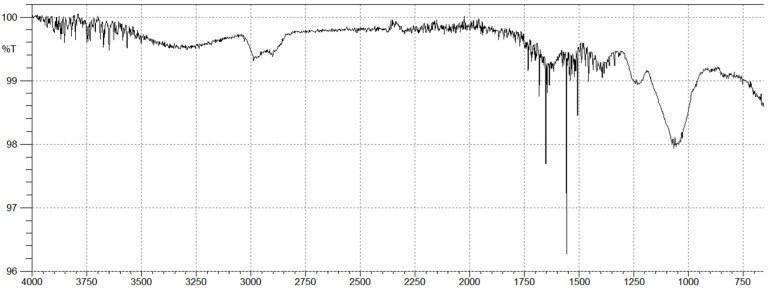
FTIR spectrum of fucoidan isolated from the Lebanese *C. sinuosa*.

Fucoidan FTIR spectrum (Figure 1) revealed that the primary absorptive peaks were a characteristic of glycosidic structures and were related to C-O and C-C stretching vibrations of the pyranoid ring (1030 cm^−1^) and the anomeric C-H group (840 cm^−1^). The FTIR spectrum of fucoidan also exhibited a broad peak at 3250–3500 cm^−1^ which was attributed to a hydroxyl stretching vibration. The two peaks at 2910 and 2989 cm^−1^ corresponded to the stretching vibration of the C-H bond of the pyranoid ring and the C-6 group of fucose. The position of sulfate groups is contained in the ranges of 1500–700 cm^−1^. Furthermore, it has been reported that the broad signal at 1240 cm^−1^ (-S-O asymmetric stretching vibration of the sulfate group) is representative of the total sulfate esters in polysaccharides [29].

**Figure 2:**
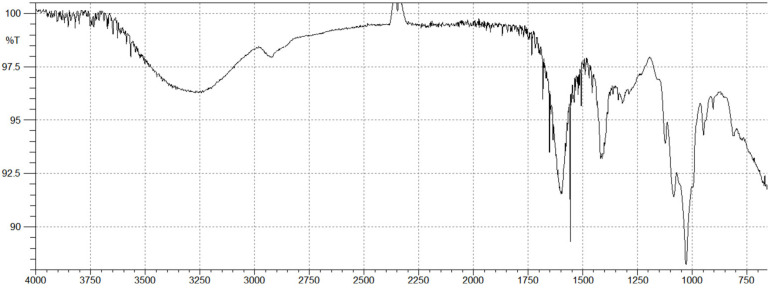
FTIR spectrum of alginate isolated from the Lebanese *C. sinuosa*.

The FTIR spectrum of alginate (Figure 2) showed a broad peak at 3280 cm^−1^ and a weak signal at 2910 cm^−1^, which are attributed to the stretching vibrations of hydroxyl groups O–H and C–H, respectively. The two strong bands at 1620 and 1400 cm^−1,^ are assigned to asymmetric and symmetric stretching vibrations of carboxyl groups of alginates. The band at 948 cm^−1^ corresponds to C–O stretching vibration of uronic acid residues and the small band at 904 cm^−1^ corresponds to C–C of α-L guluronic acid. In spectral analysis, the presence of a band at 1031 cm^−1^ was slightly more intense than the other band at 1085 cm^−1^ (M blocks), suggesting that the obtained alginate is slightly richer in guluronic residues.

### 2.3. Structural Characterization of Algal Polysaccharides by NMR Spectroscopy

**Figure 3:**
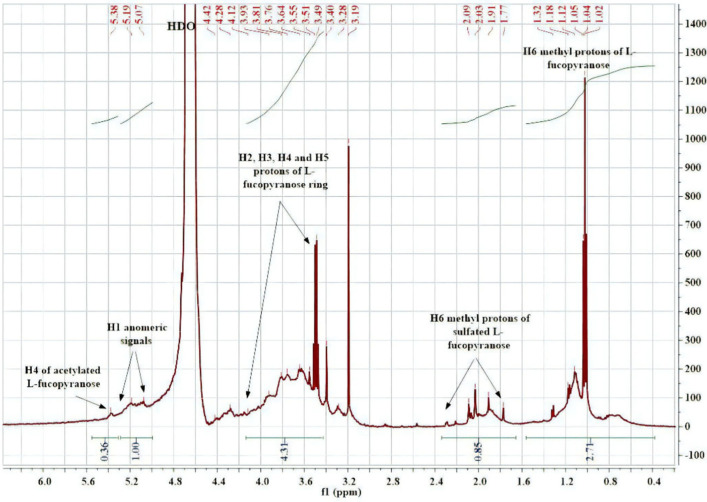
^1^H NMR spectrum of the extracted fucoidan polysaccharide.

^1^H NMR spectroscopy is the most reliable method used for the investigation of chemical composition and structural patterns. The proton spectrum of fucoidan from *C. sinuosa* (Figure 3) contained chemical shifts ranging from 5 to 5.5 ppm, which are attributed to anomeric protons (H-1) of α-linked-L-fucose and β-linked sugars. The presence of intense peaks of the methyl groups were observed between 0.8 and 1.4 ppm. This chemical shift showed the specificity of methylated protons (CH_3_) at the C-6 position of L-fucose. Monosaccharides generally do not have alkyl groups; therefore, the chemical shifts were in the range of 3.5–4.5 ppm instead of 1 ppm. The spectrum also contained resonance characteristics of L-fucopyranose ring protons (H-2 to H-5) ranging between 3.5 and 4.5 ppm. These signals confirmed the presence of different types of fucose-sulfated groups with changes in glycosidic linkage positions and monosaccharide patterns.

**Figure 4:**
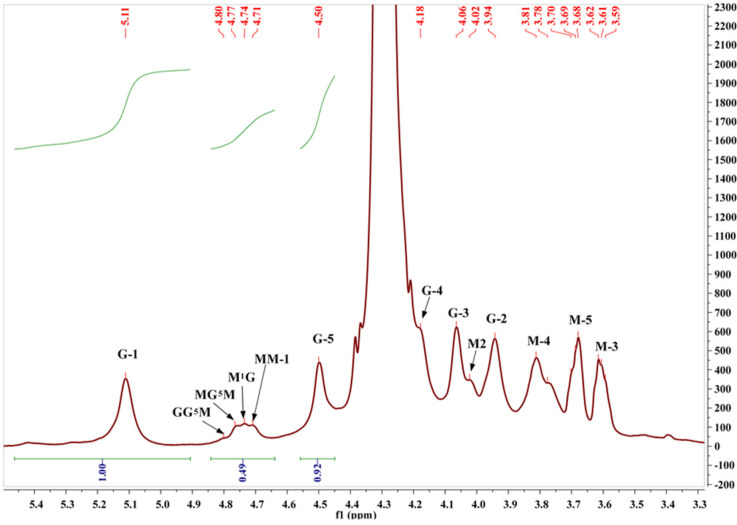
^1^H NMR spectrum of the extracted alginate.

To characterize the composition and M/G ratio of the extracted alginate, the approach of Grasdalen was adopted [30]. The relative areas of the peak I at 5.11 ppm (guluronic anomeric proton G-1), peak II at 4.71 ppm (mannuronic anomeric proton M-1 and the C-5 of alternating blocks GM-5), and peak III at 4.50 ppm (H-5 guluronic residue G-5) encompass information on the uronic acid composition and fractions of nearest neighbors along the copolymer chain [30,31,32] (Figure 4).

Numerical values for the uronic acid composition, M/G ratio, and doublet frequencies of the extracted alginate are presented in Table 3. The M/G ratio value of alginate was 0.41, indicating that alginate extracted from *C. sinuosa* has a higher fraction of guluronate in comparison to mannuronate. Additionally, alginate exhibited a higher G monomer (0.71) and dimer GG (0.65) than that of M monomer (0.29) and dimer MM (0.23) (a dimer is an oligomer consisting of two monomers joined by bonds). The ^1^H and ^13^C NMR chemical shifts of the purified alginate and fucoidan are shown in Supplementary Table S1.

### 2.4. C. sinuosa Polysaccharides Exhibited Antioxidant Activities

**Figure 5:**
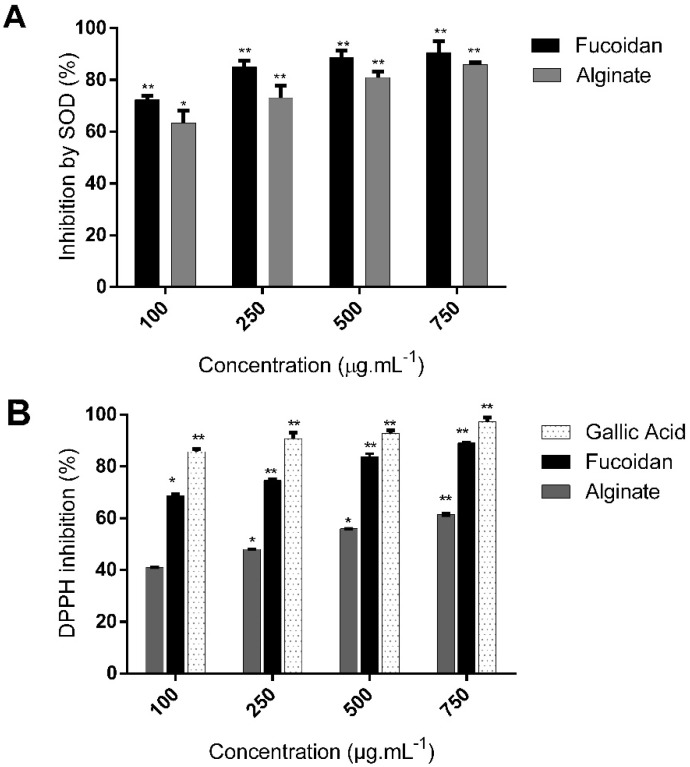
Antioxidant activity of *C. sinuosa* polysaccharides at four different concentrations (100–750 µg/mL^−1^) (**A**) Percentage inhibition of tetrazolium salts by SOD present in fucoidan and alginate (**B**) DPPH free radicals scavenging activity (% inhibition) of fucoidan and alginate (mean ± SD; *n* = 3). Significant differences are indicated as * *p* < 0.05 and ** *p* < 0.01 with respect to control.

To evaluate the antioxidant activity of isolated polysaccharides, it is recommended to use more than one assay [33]. 2,2-diphenyl-1-picryl-hydrazyl-hydrate (DPPH) assay was first used as it is an accurate, easy, and economic method to determine the radical scavenging activity of antioxidants [34]. The activity of superoxide dismutase (SOD) was also measured in the presence of fucoidan and alginate as this is one of the most important and sensitive antioxidant enzymes [35]. Fucoidan and alginate both demonstrated dose-dependent antioxidant activities, as shown in Figure 5.

Fucoidan displayed a higher antioxidant capacity than alginate in both the DPPH and SOD assays. Fucoidan treatment increased the antioxidant SOD activity, as evidenced by the inhibition of water-soluble tetrazolium salt, to a greater extent (90.7 ± 4.4% at 750 µg/mL^−1^) than alginate (86 ± 0.7%) (Figure 5A). The SOD IC_50_ value of fucoidan was lower than that of alginate (23.7 ± 1.1 and 41.34 ± 1.07 µg/mL^−1^, respectively). We then compared the DPPH scavenging activity of fucoidan and alginate to the known antioxidant compound gallic acid (Figure 5B). Fucoidan possessed a significantly higher scavenging activity (89% ± 0.4% at 750 µg/mL^−1^) than alginate (61.6 ± 0.5%), and slightly lower activity than gallic acid (97.2 ± 1.6%). The DPPH IC_50_ value of fucoidan was also lower than that of alginate (46.2 ± 1.4 and 280 ± 1.2 µg/mL^−1^, respectively).

### 2.5. C. sinuosa Polysaccharides Showed Potent Antitumor and ROS-Inducing Effects against HCT-116 Colon Cancer Cells

**Figure 6:**
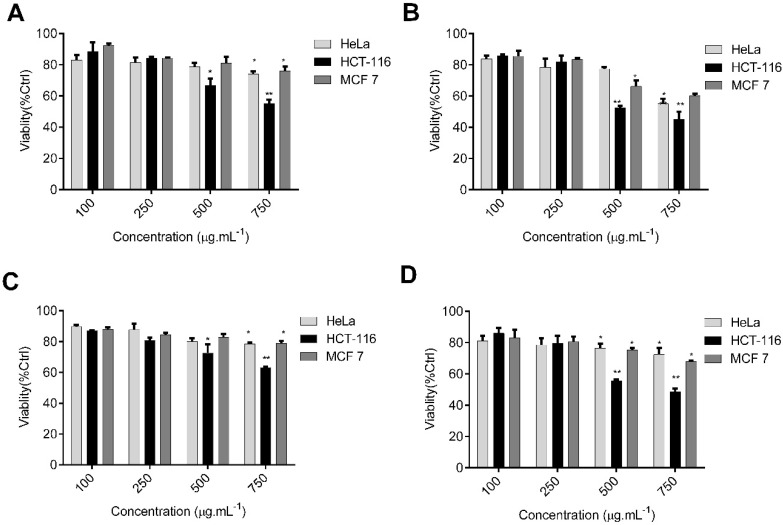
Cytotoxic effects of fucoidan and alginate on the viability of various cancer cells (HCT-116, MCF 7, and HeLa). Different concentrations of fucoidan on tumor cells were tested at 24 h (**A**) and 48 h (**B**). Cytotoxic potentials of alginate against these cancer cell lines were also examined at 24 h (**C**) and 48 h (**D**) (mean ± SD; *n* = 3). Significant differences are indicated as * *p* < 0.05 and ** *p* < 0.01 with respect to control.

First, we examined the antitumor properties of the extracted polysaccharides by exposing colon HCT-116, cervical HeLa, and breast MCF 7 cells to different concentrations, and then determined their effect on cell viability using MTT assay. We observed dose and time inhibitory effects of both compounds on cell viability at 24 and 48 h (Figure 6).

There was a significant decrease in cell viability in HCT-116 cells treated for 24 h at high concentrations of fucoidan (45% inhibition at 750 µg/mL^−1^, Figure 6A) in comparison to low concentrations (11.5% cell inhibition at 100 µg/mL^−1^). Similar dose-dependent inhibition patterns were observed upon treatment with alginates, with 37.1% inhibition observed at 750 µg/mL^−1^ (Figure 6C). The viability of HCT-116 colon cancer cells treated with 750 µg/mL^−1^ of fucoidan at 48 h was lower than MCF 7 and the HeLa cell lines (Figure 6B). Similar results were obtained with alginate treatments on the selected cancer cell lines (Figure 6D). Thus, the HCT-116 cell line was the most sensitive to the polysaccharides, with fucoidan having an IC_50_ value (618.9 µg/mL^−1^) lower than that of alginate (690 µg/mL^−1^) (Supplementary Table S2).

**Figure 7:**
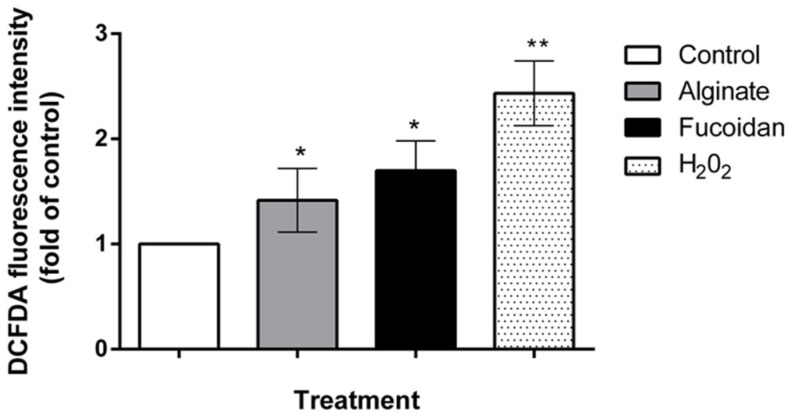
Fucoidan and alginate isolated from *C. sinuosa* trigger an increase in DCFDA fluorescence intensity, reflecting the enhanced ROS levels in HCT-116 colon cancer cells (mean ± SD; *n* = 3). Significant differences are indicated as * *p* < 0.05 and ** *p* < 0.01 with respect to control.

The induction of apoptosis by ROS is considered to be a central mechanism in cancer therapy, and several recent approaches for colon cancer treatment are based on modulating ROS levels [36]. To investigate mechanisms involved in cell death, we used the fluorescent probe DCFDA to quantify ROS production in the most sensitive cell line HCT-116 upon treatment with fucoidan and alginate. As shown in Figure 7, treatment with fucoidan and alginate triggered a significant generation of intracellular ROS in comparison to control, suggesting that the induction of ROS is an important event in the cell death mechanism caused by these marine polysaccharides.

### 2.6. Combination Treatment of Vitamin C and C. sinuosa Polysaccharides Significantly Increase the Cytotoxic Activity against HCT-116 Cancer Cells

**Figure 8:**
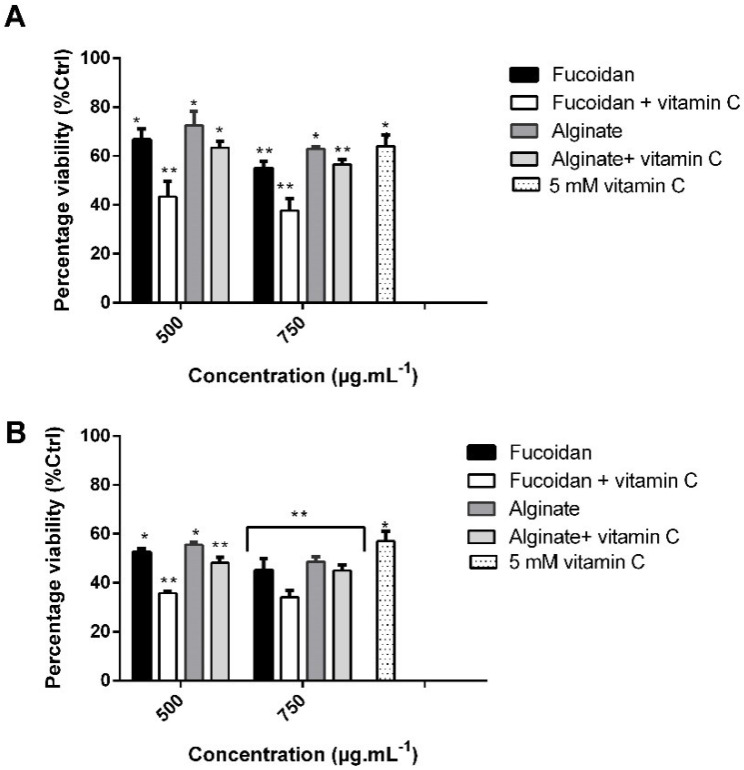
Influence of polysaccharides from *C. sinuosa* at two different concentrations and their combination with vitamin C on HCT-116 cell viability after (**A**) 24 h and (**B**) 48 h incubation (mean ± SD; *n* = 3). Significant differences are indicated as * *p* < 0.05 and ** *p* < 0.01 with respect to control.

Since the purified fucoidan and alginate of *C. sinuosa* were mostly effective against HCT-116 cells, we assessed their effects at two different concentrations (500 and 750 µg/mL^−1^) in combination with 5 mM vitamin C at 24 and 48 h. We tested whether the combined treatments resulted in enhanced cytotoxicity with respect to single treatments (Figure 8A,B).

As shown in Figure 8B, the combination of 5 mM vitamin C with 750 µg/mL^−1^ of fucoidan at 48 h resulted in a significant reduction in cell viability (34.2%), in comparison to either compound alone (45 and 57% decrease in presence of fucoidan or vitamin C, respectively). The decrease in HCT-116 cell viability when treated with alginate and vitamin C was almost similar (45% at 48 h) to alginate treatment alone (48.6%). Thus, the combination of vitamin C with fucoidan appears to display a more potent antitumor effect than combining vitamin C with alginate.

### 2.7. Combination of Vitamin C and Algal Polysaccharides Trigger Cell Cycle Regulation and Morphological Alterations in HCT-116 Cells

**Figure 9:**
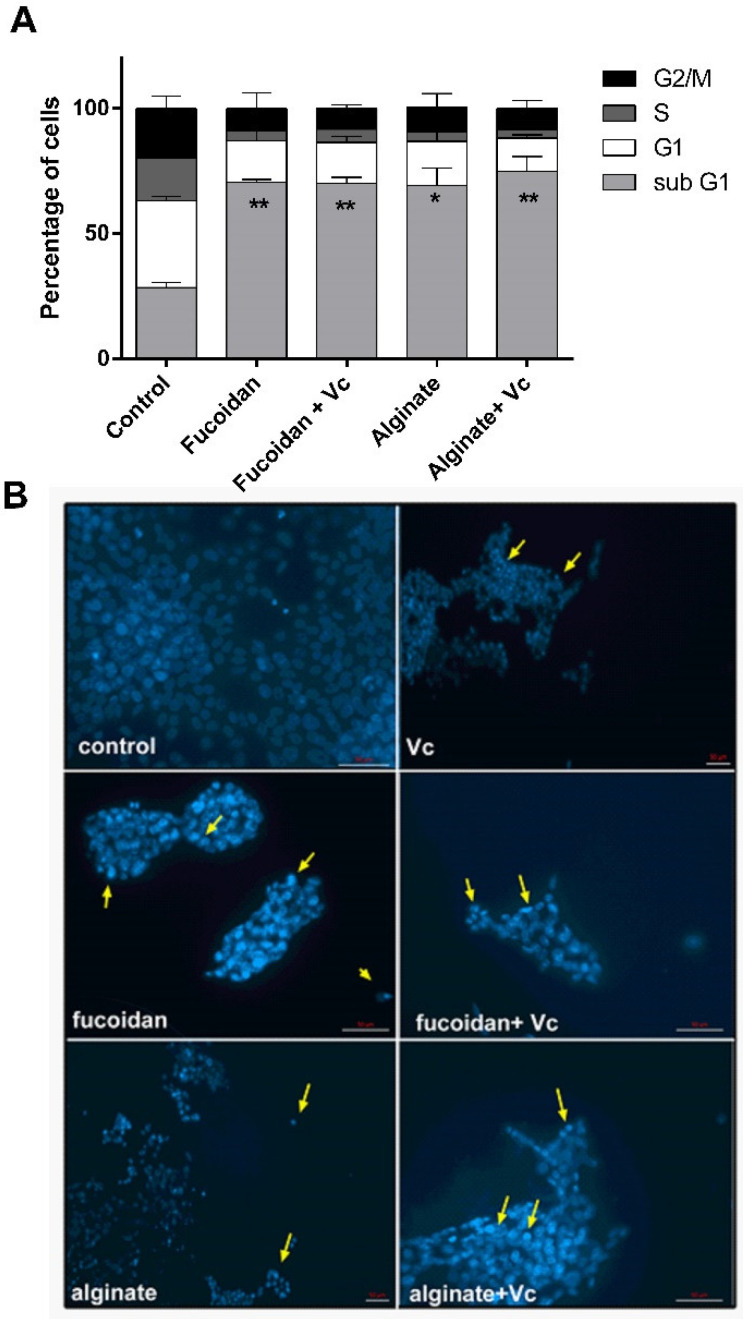
Cell cycle and morphological analysis of HCT-116 colorectal cancer cells treated with fucoidan, alginate, and their combination with vitamin C (**A**) Flow cytometry analysis shows the effects of fucoidan, alginate, and their combination on the cell cycle progression at 24 h; (**B**) DAPI micrographs of HCT-116 cells indicated that nuclear fragmentation and chromatin condensation occurred in vitamin C, fucoidan, and alginate-treated cells alone or with vitamin C combination. Nuclei were counterstained with DAPI (blue), scale bars = 50 μm. Significant differences vs. control cells, comparing cell cycle phases (SubG1, G0/G1, S, G2/M), are indicated as * *p* < 0.05 and ** *p* < 0.01 with respect to control.

To examine whether the inhibition of viability by the combination treatment was due to cell cycle arrest and/or apoptosis, cell cycle analysis was performed using propidium iodide (PI) staining of DNA followed by flow cytometry. Treatment of HCT-116 cells with fucoidan and alginate alone or in combination with vitamin C yielded a significant accumulation of cells in the sub G1 phase of the cell cycle in comparison to control (28.5%) (Figure 9A). 

The number of cells in the G1 phase decreased significantly from 35% in control to almost 17% after treatment with fucoidan alone or its combination with vitamin C (Figure 9A). The percentage of cells in the G1 phase also decreased post alginate treatment, particularly when alginate was combined with vitamin C. These results indicate that fucoidan and alginate (alone or in combination with vitamin C) induce cell death in HCT-116 cancer cell line. Separation and gating of the different phases of the cell cycle are given in Figure S3.

To investigate the cell death mechanism by fucoidan and alginate, we analyzed the morphological changes observed in the nuclei of treated HCT-116 cells using DAPI staining (Figure 9B). Apoptosis is known to cause different morphological changes in cells, which include cell shrinkage, cytoplasm, nuclear and chromatin condensation, and nuclei degradation into discrete particles [37]. Apoptotic features were observed in microscopic images of HCT-116 cells after 24 h of treatment with 750 µg/mL^−1^ fucoidan, alginate, and vitamin C (5 mM) combinations. Both polysaccharides caused cellular changes which included cell shrinkage, discrete particles, and chromatin and cytoplasmic condensations (Figure 9B). Thus, flow cytometry analysis and DAPI staining both suggested the possible induction of apoptosis in HCT-116 cells in response to fucoidan and alginate treatment.

## 3. Discussion

Marine polysaccharides have numerous anticancer properties that make them interesting candidates for integration in drug discovery and biomedical applications [38]. In our previous study, the elemental, organic composition, and phenolic content of *C. sinuosa* extract was determined [28]. The apoptotic effects of the crude phenol-rich extracts of this algae were then tested against colon cancer [39] without characterizing the chemical structure of the components. Considering that the polysaccharides of *C sinuosa* are the most abundant components [28], we hereby characterized the fucoidan and alginate polysaccharides present in this algae and studied their anticancer activity alone and in combination with vitamin C against a panel of solid tumors. Alginates and fucoidans are known to be safe to normal cells, as they are also available in dietary supplements [40]. To our knowledge, fucoidans and alginates extracted from *C. sinuosa* and their combination with vitamin C have never been evaluated for their anticancer activity. Previous studies documenting the cytotoxic effects of polysaccharides from Lebanese brown algae were only based on MTT assays rather than on mechanistic biological approaches that could allow meaningful comparisons. Thus, this is the first report that documents the use of the Lebanese macroalgal polymers as natural medicinal sources of anticancer biomolecules in combination with vitamin C.

Since structural variations play a major role in determining the biological properties of a polymer, we first studied the structure of the isolated polysaccharides from the Lebanese *C. sinuosa* [10]. The FTIR spectra revealed two strong bands at 1620 and 1400, assigned to asymmetric and symmetric stretching vibrations of carboxyl groups of alginates. The anomeric region of this carbohydrate, between 950 and 750 cm^−1^, is the most discussed in the literature [41]. The presence of a band at 815 cm^−1^ in the alginate spectrum is attributed to mannuronic acid residues [42]. The guluronic units showed a band at approximately 1031 cm^−1^, higher than that of mannuronic units at 1085 cm^−1^ [43].

It is also well known that sodium alginate is a linear macromolecule composed of poly-β-1, 4-d-mannuronic acid (unit M) and α-1,4-l-glucuronic acid (unit G) [44]. Interestingly, the alginates isolated from *C. sinuosa* were richer in guluronic than mannuronic acid, indicating that these alginates can form strong and heat-stable hydrogels. This is because the guluronate blocks allow a high degree of divalent ion coordination, which is essential for enhancing gelling properties [45]. However, it must be noted that the structure of the alginate extracted from the Lebanese *C. sinuosa* is slightly different from alginates present in brown algae of other species in the same region. For instance, the alginate we extracted had lower M/G ratio (0.41) than those extracted from *Sargassum vulgare* (0.78) and *Stypopodium schimperi* (0.96) [46,47]. Additionally, most fucoidans of brown seaweeds consist mainly of sulfated L-fucose (about 34–44%) [48], which is in parallel with our physicochemical analysis showing the elevated levels of fucose. The structure of fucoidan was further validated by the IR spectra indicating that the isolated fucoidan had close structural similarity to commercial fucoidan [49,50]. The profiles of the proton NMR spectrum of fucoidans from *C. sinuosa* were similar to several other fucoidans extracted from different origins and were comparable to fucoidans reported in *Stypopodium schimperi* growing on the Lebanese coast [47,51,52,53].

Establishing consistent bioactivities for fucoidan and alginate is a challenge because of variations in extraction methods, species-related structural diversity, growth conditions, harvest season, among other factors [54]. To a large extent, the bioactivity of these biopolymers is correlated with their structural characteristics. Alginate is recognized as an ideal candidate for chemical functionalization, and this is mainly due to the free hydroxyl and carboxyl groups distributed along the backbone, which allows the polymer to be modified and improve physicochemical and biological features [55]. It is known that the antioxidant potential of acidic polysaccharides is strongly related to their uronic acid content [56]; thus, the strong antioxidant activity of the extracted alginate could be attributed to their higher uronic acid content. Based on the literature, a higher sulfation degree has been tightly correlated with augmented bioactivity responses, including antioxidant potential [57]. The sulfate content of *C. sinuosa* fucoidan (18.8%) appeared to be higher than that of commercial fucoidan (14.4%), sargassum (4.7%), and padina (8.8%), but slightly lower than that of turbinaria species (19.4%) [58]. Thus, the stronger antioxidant potential of fucoidans of *C. sinuosa* could be attributed to their higher sulfate content. When comparing the isolated fucoidan in this study to those extracted from different algal species elsewhere, we found that fucoidans of *C. sinuosa* had the highest DPPH scavenging potential [59,60,61,62].

Previous in vitro and in vivo studies have shown that algal polysaccharides exert antitumor effects, and suppress growth, angiogenesis, and metastasis in a range of cancer cells [63]. The cytotoxic potential of fucoidan and alginates isolated from *S. schimperi* revealed lower cytotoxicity on colon cancer cell lines compared to extracts from *C. sinuosa* [47]. Moreover, fucoidans of Lebanese origin isolated from *Dictyopteris* and *Sargassum* species exerted significant cytotoxic potential on human melanoma cells [64]. In this study, alginate and fucoidan of *C. sinuosa* were tested against different types of solid tumors, and the colon cancer HCT-116 cell line was found to be the most sensitive.

Furthermore, the ROS levels in HCT-116 cells were elevated upon treatment with polysaccharides from *C. sinuosa*. This increase in ROS was more pronounced upon fucoidan treatment (1.7-fold). This is in accordance with other studies that have shown that fucoidans from different algal species induce apoptosis in cancer cells via ROS-mediated cell signaling [65,66,67,68]. In addition, the combination of alginate with vanadyl [69] or with tamoxifen [70] was found to induce ROS-dependent cell death in cancer cells.

Many investigations have highlighted the importance of marine polysaccharides as candidates for combination therapy [8,71]. However, so far, only one study has investigated the effect of a mixture of dietary fucoidan from Japanese Mozuko seaweed with sodium ascorbic acid, and the results confirmed that this combination had strong antioxidant activity which was associated with an enhanced inhibition of fibrosarcoma tumor invasion in vitro [72]. In our study, the combination of vitamin C with fucoidan and alginates (IC_50_ 437.4 and 453.3 µg/mL^−1^, respectively) acted synergistically to significantly increase cytotoxicity against colorectal cancer cells. This combination significantly decreased the IC_50_ of the isolated fucoidan to lower levels than fucoidans isolated from other regions and sources [6].

To unravel the mechanisms of cell death induced by the polysaccharides of *C. sinuosa*, their effects on the morphology and cell cycle regulation of HCT-116 cells were evaluated by flow cytometry and DAPI staining. Apoptosis is a major control mechanism in living organisms, and its deregulation results in cancer development; thus, the induction of apoptotic cell death in cancer cells is an effective strategy for cancer cell eradication [73]. Fucoidan, alginate, and their combination with vitamin C revealed a significant increase in the proportion of cells present in the sub G1 phase. This reflects a typical cell death pattern [74], which was confirmed to be apoptosis by the morphological changes and apoptotic features revealed by DAPI staining. Other studies have shown that fucoidans extracted from different origins induced similar cell cycle alterations in MCF 7, HCT-116, and Caco cancer cell lines, as evidenced by the increase in the proportion of cells in the sub G1 phase and apoptosis induction via ROS-dependent mechanisms [68,75,76].

In conclusion, this study provides new insights for the use of marine polysaccharides from Lebanese brown algae as natural medicinal sources. Fucoidan and alginates from *C. sinuosa* appear to have promising antitumor activity against different human cancer cell lines. Their combination with vitamin C caused enhanced cell cycle alterations, ROS generation, and apoptotic cell death in HCT-116 colon cancer cell line. Henceforth, fucoidans and alginates could be used in combination with vitamin C as dietary supplements or as adjuvant treatments against cancer.

## 4. Materials and Methods

### 4.1. Sample Collection

*C. sinuosa* samples were collected in July, from the Al Qalamoun area of the North Lebanese coast of the Mediterranean at a depth of 3–5 m. Fresh seaweed was rinsed, air dried, and ground to a fine powder. A voucher specimen (No.20181102A) was deposited in the Doctoral School of Science and Technology, Lebanese University, Tripoli, Lebanon.

### 4.2. Extraction of Polysaccharides from C. sinuosa

*C. sinuosa* was extracted in absolute ethanol overnight in order to remove pigments, fatty acids, and oligoelements. The mixture was centrifuged at 3000 rpm for 20 min, and the residue was washed thoroughly with ultrapure water. This residue was re-extracted with diluted hydrochloric acid (HCl 0.01 M) twice at 60 °C for 3 h for further depigmentation and centrifuged at 3000 rpm for 20 min. The supernatant (1) obtained was used for fucoidan extraction, while the residue (2) was used for the isolation of alginates [47].

#### 4.2.1. Fucoidan Isolation

The supernatant (1) was neutralized, rotavapored, and then dialyzed overnight to remove impurities using dialysis cellulose tubing (Sigma, St. Louis, MO, USA, MW cutoff: 1200 KDa). Dilute HCl was added to precipitate mannularin and the hydrolysate was centrifuged (3000 rpm, 20 min). The obtained residue was discarded, and the supernatant containing the crude fucoidan was lyophilized [47].

#### 4.2.2. Alginate Isolation

Residue (2) was extracted with sodium bicarbonate (Na_2_CO_3_) for 8 h, diluted to 1.5% Na_2_CO_3_, and extracted for another 8 h. After centrifugation, the supernatant was rotavapored and dialyzed overnight. Alginic acid was precipitated with ethanol (1:1, *v/v*) and again centrifuged to obtain the pellet of interest. This pellet was then dissolved in ultrapure water, and 5 drops of 0.01 M HCl was added. Sodium alginate was obtained by adding a few drops of NaOH to reach pH 8. This process was followed by dialysis and lyophilization to obtain sodium alginate [47].

#### 4.2.3. Fucoidan and Alginate Purification

The purification step was adopted and slightly modified from Sari-Chmayssem et al. (2016). Briefly, fucoidan and alginate were dissolved in 0.3 M HCl and heated at 50 °C for 3 h. After cooling, the mixture was centrifuged at 5000 rpm for 10 min and the supernatant was neutralized with 1 M NaOH and poured over 10 mL of ethanol. Finally, the precipitate was dissolved and lyophilized [46].

### 4.3. Physicochemical Properties

The yield of each purified polysaccharide was determined by weighing the mass after purification and lyophilization. Next, the total phenolic content (TPC) of each polysaccharide was estimated using the Folin-Ciocalteu method [77]. The absorbance values of polysaccharides were compared with gallic acid standard. The TPC was expressed as mg of gallic acid equivalents (GAE) per gram of powder on a dry weight (DW) basis. The percentage of proteins was estimated using the DC protein Lowry method according to the manufacturer instructions (BioRad kit), with bovine serum albumin used as a standard. The sulfate content was further determined turbidimetrically by adopting the barium chloride-gelatin method and potassium sulfate as a standard [78]. The glucuronic acid content was assessed spectrophotometrically using D-glucuronic acid as the standard [79].

### 4.4. Monosaccharide Composition

For the determination of monosaccharide content by HPLC, fucoidan and alginate were hydrolyzed with 2 M trifluoroacetic acid (TFA) at 121 °C for 4 h in glass tubes sealed under nitrogen air. After reaction, the liquid fraction was neutralized to pH 7 and then dried under nitrogen air flow. Samples were then injected into the HPLC system, equipped with a refraction-index (RI) detector. LC-NH_2_ column (Sigma) was used for the chromatographic separation of reducing sugars [80]. Glucose, galactose, fucose, mannose, xylose, and arabinose (Sigma) were used as standards.

### 4.5. Fourier Transform Infrared Spectroscopy (FTIR) Analysis

FTIR spectra of fucoidan and alginate were acquired using a SHIMATZU instrument (MIRacle 10 series, total reflectance method). For this analysis, the samples with no additional treatments (2 mg) were analyzed. The frequency of the spectra set to analysis was between 4000 and 150 cm^−1^ wave number, and the vibration spectra were recorded graphically. The M/G ratio of alginate was estimated from specific absorption bands at approximately 1030 and 1090 cm^−1^ [42,81].

### 4.6. Nuclear Magnetic Resonance (NMR) Spectroscopy

^1^H and ^13^C NMR spectra of alginate and fucoidan were recorded at 70 °C on a Bruker500 MHz spectrometer. Lyophilized samples of fucoidan and alginate (10 mg, pH 7) were dissolved in deuterium oxide (D_2_O).

G and M quantitative analysis in alginate:

Quantitatively, the mole fraction of G and the doublet frequency F_GG_ are related to the areas (A) of the respective peaks (I, II, and III) by the following relationships [46]:

(1) $$\mathrm{FG}=\frac{\mathrm{AI}}{\mathrm{AII}+\mathrm{AIII}}$$

(2) $$\mathrm{FGG}=\frac{\mathrm{AIII}}{\mathrm{AII}+\mathrm{AIII}}$$

The mole fraction of M was then calculated from the equation:

F_G_ + F_M_ = 1 (3)

The M/G ratio is given by the following:

(4) $$\frac{M}{G}=\frac{(1\;-\;\mathrm{FG})\;}{\mathrm{FG}}$$

Doublet frequencies (F_GG_ and F_MM_) were obtained from the following equations:

F_G_ = F_GG_ + F_GM_ (5)

F_M_ = F_MM_ + F_MG_ (6)

For high-molecular-weight alginate (DP > 20), we considered that:

F_MG_ = F_GM_ (7)

### 4.7. Preparation of Polysaccharides

Ultrasound sonication is an effective approach to decrease the viscosity of isolated polysaccharides with minor structural destruction [82]. To reduce the viscosity and molecular weight of the isolated polysaccharides from *C. sinuosa*, fucoidan and alginate stock solutions were prepared and sonicated. They were then dissolved in phosphate buffer saline (PBS 1X) and sonicated in a water bath (Bioruptor^®^ Plus sonication) for 3 h at 50 °C.

### 4.8. Antioxidant Assays

#### 4.8.1. DPPH Free Radical Scavenging Assay

The DPPH antioxidant assay is based on the reduction of DPPH in the presence of a proton-donating compound and is used to evaluate the antioxidant activity of natural compounds [83]. The scavenging effects of samples for DPPH radical were monitored according to the method of a previous report [84]. Fucoidan and alginate were aliquoted into a range of concentrations (100, 250, 500, and 750 µg/mL^−1^). The absorbances were measured at 517 nm. All tests were performed in triplicates.

#### 4.8.2. Superoxide Dismutase (SOD) Inhibition Assay

SOD is an important antioxidative enzyme which catalyzes the dismutation of the superoxide anion (O_2_^−^) into hydrogen peroxide and oxygen. SOD activity represented the percentage of the inhibition of water-soluble tetrazolium salt (WST-1) [85]. This inhibition was measured in fucoidan and alginate according to the manufacturer instructions using a commercial kit of SOD (19160) (Sigma-Aldrich, St. Louis, USA), and the absorbances were determined at 450 nm. All tests were performed in triplicates.

### 4.9. Cell Lines and Culture

Human colon cancer (HCT-116), human breast cancer (MCF 7), and human cervical cancer (HeLa) cell lines were purchased from the American Type Culture Collection (ATCC). Cells were cultured in DMEM at 37 °C in a humidified atmosphere of 5% CO_2_ and 95% air. Media were supplemented with 1% Penicillin Streptomycin (100 µg/mL^−1^) and 10% heat-inactivated fetal bovine serum (FBS).

### 4.10. Cell Viability Assay

The cell growth assay is a dimethyl thiazolyldiphenyltetrazolium (MTT)-based method that measures the ability of metabolically active cells to convert tetrazolium salt into a blue formazan product [86]. Cells were seeded in a 96-well plate at a density of 10^4^ overnight and then cells were treated with various concentrations of fucoidan and alginate (100–750 µg/mL^−1^). After 24 and 48 h, treatments were removed, and cells were washed prior to MTT incubation for 2 h at 37 °C. The mean absorbance values of three experiments were expressed as a percentage of viability relative to the control untreated cells. The most sensitive cell line was further subjected to another MTT experiment to test fucoidan and alginate combinations with 5 mM vitamin C. The absorbance was recorded at 570 nm.

### 4.11. Quantitative Determination of ROS

Intracellular ROS generation was quantified using CM-H2DCFDA. This method is based on the formation of highly fluorescent 2′,7′-dichlorofluorescein (DCF) from non-fluorescent CM-H2DCFDA. Hydrogen peroxide (20 µM H_2_O_2_) was used as a positive control to induce ROS production. Cells were treated with alginate and fucoidan at a concentration of 750 µg/mL^−1^ and incubated for 4 h at 37 °C. At the end of the treatment period, cells were incubated with 20 µM of dye, in a serum and phenol red free medium for 30 min. The fluorescence intensity was detected using a microplate fluorometer TriStar2S LB942 (Brethold, Bad Wildbad, Germany) at an excitation wavelength of 485 nm and an emission wavelength of 528 nm.

### 4.12. Cell Cycle Analysis

Dead and viable cells were collected 24 h post-treatment with 750 µg/mL^−1^ of the algal polysaccharides and 5 mM vitamin C combinations. The pellets were washed with ice-cold PBS, fixed with 70% ice-cold ethanol, and stored at −20 °C overnight. Cells were then washed twice with PBS and incubated with 200 µg/mL^−1^ of RNAse A for 1 h at 37 °C before staining with 0.625 µg/mL^−1^ of PI for 30 min. The fluorescence intensity was measured by flow cytometry using a fluorescence-activated cell sorter (FACS) and analyzed using Cell Quest.

### 4.13. DAPI Staining

Morphological changes of the nuclei of treated cells were investigated under a confocal laser scanning microscope using DAPI staining. Briefly, the cells were treated with 750 µg/mL^−1^ of fucoidan, alginate, and their combination with vitamin C. After 24 h, cells were washed with 1× PBS, then fixed and stored overnight at −20 °C. Cells were stained with DAPI in the dark. DAPI-stained cells were photographed with a fluorescence microscope using a blue filter (40 and 20× magnifications).

### 4.14. Statistical Analysis

All statistical analysis (*t*-test and one-way ANOVA) were performed using GraphPad Prism 7 (version 7.0, GraphPad Software Inc., San Diego, CA, USA). Probability values below 0.05 (* *p* < 0.05) were considered significant and values below 0.01 (** *p* < 0.01) were considered highly significant. All quantitative variables were reported as mean ± SD.

## Figures and Tables

**Table 1:** Physicochemical characteristics of the purified fucoidan and alginate isolated from *C. sinuosa*.

Sample	Yield (%)	Protein (%)	Sulfate (%)	Phenol (mg GAE/gDW)	D-Glucuronic Acid (%)
Fucoidan	11.6 ± 0.3	1.88 ± 1.1	18.8 ± 2.1	0.045 ± 0.01	3.8 ± 0.5
Alginate	13.6 ± 0.4	2.63 ± 1.4	5.53 ± 2.6	0.043 ± 0.01	50 ± 0.7

Values are presented as mean ± SD (*n* = 3); DW: dry weight; GAE/gDW: gallic acid equivalent per gram of dry weight.

**Table 2:** Monosaccharides composition of fucoidan and alginate of *C. sinuosa* determined by HPLC-RI analysis.

Sample	Monosaccharide Composition (%)					
	Glucose	Xylose	mannose	Arabinose	Galactose	fucose
Fucoidan	5.45 ± 2.97	2.62 ± 2.12	3.58 ± 1.34	3.4 ± 1.39	5.94 ± 3.88	67.4 ± 12.1
Alginate	2.67 ± 3.59	2.14 ± 2.48	5.11 ± 2.64	4.2 ± 3.67	4.98 ± 4.66	0.2 ± 0.34

Values are presented as mean ± SD (*n* = 3).

**Table 3:** Numerical values for the uronic acid composition, M/G ratio, and doublet frequencies of alginate extracted from *C. sinuosa*.

Numeric Values	F_G_	F_M_	M/G Ratio	F_GG_	F_GM_ = F_MG_ *	F_MM_
Alginate	0.71	0.29	0.41	0.65	0.06	0.23

* F_GM_ refers to the frequency of the dimer formed by guluronate-mannuronate; F_GM_ refers to the frequency of the dimer formed by mannuronate-guluronate.

## Data Availability

All data and materials support our published claims and comply with field standards. Original data could be made available upon request.

## Supplementary Materials

Figure S1: Alginate monosaccharide spectrum by HPLC analysis coupled to RI detector, Figure S2: Fucoidan monosaccharide spectrum by HPLC analysis coupled to RI detector, Figure S3: Cell cycle gating of HCT-116 treated with fucoidan and alginate isolated from C. sinuosa, together with their combinations with vC, Table S1: ^1^H and ^13^C NMR chemical shifts of purified (A) alginate and (B) fucoidan recorded at 70 °C, Table S2: Fucoidan and alginate IC50 against different cancer cell lines (HCT-116, MCF7, and HeLa) at 24 and 48 h.

## Sample Availability

Samples of the compounds fucoidan and alginateare available from the authors.
