# Supplementary material for: Fucoidan and Alginate from the Brown Algae Colpomenia sinuosa and Their Combination with Vitamin C Trigger Apoptosis in Colon Cancer
Source: Molecules. 2022 Jan 6;27(2):358. doi: 10.3390/molecules27020358 (PMC8777791; doi:10.3390/molecules27020358)
Supplement: Supplementary file 1 [file molecules-27-00358-s001.zip › molecules-1527018-supplementary.pdf]

Fucoidan and Alginate from the Brown Algae *Colpomenia sinuosa* and Their Combination with Vitamin C Trigger Apoptosis in Colon Cancer

Reem Al Monla [1], Zeina Dassouki [1], Nouha Sari-Chmayssem [1], Hiba Mawlawi [1] and Hala Gali-Muhtasib [2,3,*]

Table S1. ^{1}H and ^{13}C NMR chemical shifts of purified (**A**) alginate and (**B**) fucoidan recorded at 70 °C.

**A. Alginate**	1	2	3	4	5	6
^{1}H NMR						
G	5.11	3.94	4.06	4.18	4.50	-
M	4.71	4.02	3.57-3.63*	3.74-3.87*	3.65-3.72*	-
^{13}C NMR						
G	101.20	65.74	69.72	80.45	67.91	175.60
M	100.59	70.45		72.67	76.69	
**B. Fucoidan**						
^{1}H NMR	5.07-5-19	H2 to H-5 : 3.4-4.2 ppm				1.02-1.32
^{13}C NMR	95	C2 to C5 : 65-83 ppm				20

*Intervals of δ ppm corresponding to the presence of several peaks relative to different isomers of mannuronate.

Table S2. Fucoidan and alginate IC_{50} against different cancer cell lines (HCT-116, MCF7, and HELA) at 24 and 48 h.

Concentration (µg.mL^{-1})	HCT-116	MCF 7	HeLa
Fucoidan 24 h	929.2±1.14	5197±1.21	4964±1.18
Alginate 24 h	1725±1.23	5410±1.14	9918±1.24
Fucoidan 48 h	618.9±1.99	1247±1.54	1434±2.31
Alginate 48 h	691±1.12	4994±1.27	6021±1.11

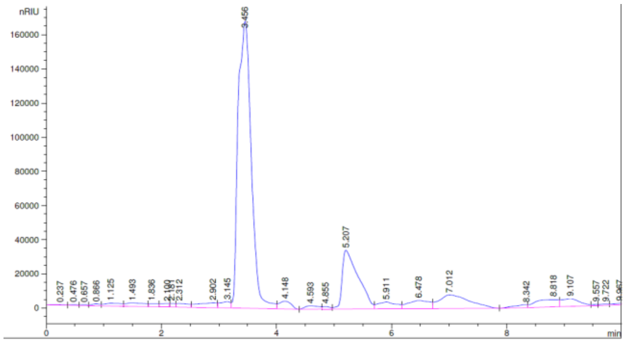

Figure S1. Alginate monosaccharide spectrum by HPLC analysis coupled to RI detector.

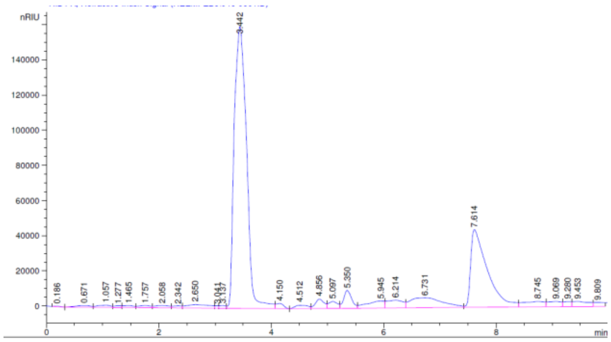

Figure S2. Fucoidan monosaccharide spectrum by HPLC analysis coupled to RI detector.

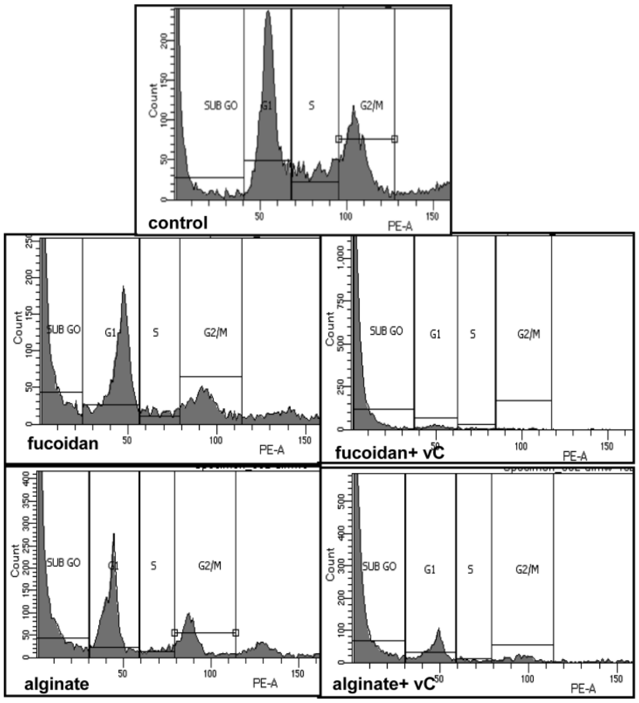

Figure S3. Cell cycle gating of HCT-116 treated with fucoidan and alginate isolated from *C. sinuosa*, together with their combinations with vC.
